# Halophilic Archaea *Halorhabdus Rudnickae* and *Natrinema Salaciae* Activate Human Dendritic Cells and Orient T Helper Cell Responses

**DOI:** 10.3389/fimmu.2022.833635

**Published:** 2022-05-26

**Authors:** Krzysztof T. Krawczyk, Camille Locht, Magdalena Kowalewicz-Kulbat

**Affiliations:** ^1^Department of Immunology and Infectious Biology, Institute of Microbiology, Biotechnology and Immunology, Faculty of Biology and Environmental Protection, University of Lodz, Lodz, Poland; ^2^Univ. Lille, CNRS, Inserm, CHU Lille, Institut Pasteur de Lille, U1019 – UMR9017 – CIIL – Center for Infection and Immunity of Lille, Lille, France

**Keywords:** halophilic archaea, Halorhabdus rudnickae, Natrinema salaciae, dendritic cells, T cells

## Abstract

Halophilic archaea are procaryotic organisms distinct from bacteria, known to thrive in hypersaline environments, including salt lakes, salterns, brines and salty food. They have also been identified in the human microbiome. The biological significance of halophiles for human health has rarely been examined. The interactions between halophilic archaea and human dendritic cells (DCs) and T cells have not been identified so far. Here, we show for the first time that the halophilic archaea *Halorhabdus rudnickae* and *Natrinema salaciae* activate human monocyte-derived DCs, induce DC maturation, cytokine production and autologous T cell activation. *In vitro* both strains induced DC up-regulation of the cell-surface receptors CD86, CD80 and CD83, and cytokine production, including IL-12p40, IL-10 and TNF-α, but not IL-23 and IL-12p70. Furthermore, autologous CD4^+^ T cells produced significantly higher amounts of IFN-γ and IL-13, but not IL-17A when co-cultured with halophile-stimulated DCs in comparison to T cells co-cultured with unstimulated DCs. IFN-γ was almost exclusively produced by naïve T cells, while IL-13 was produced by both naïve and memory CD4^+^ T cells. Our findings thus show that halophilic archaea are recognized by human DCs and are able to induce a balanced cytokine response. The immunomodulatory functions of halophilic archaea and their potential ability to re-establish the immune balance may perhaps participate in the beneficial effects of halotherapies.

## Introduction

Archaea represent a separate domain of life, with unique genetic and biochemical features, distinct from Eukaryotes and bacteria (1, 2). Initially, archaea were described as extremophiles – organisms able to live in extreme environmental conditions, ranging from artificial solar salterns, to natural brines in coastal and submarine pools and salt mines, high/low temperature, pressure, dryness, radiation, pH, concentration of heavy metals or salinity. Halophiles are the habitants of hypersaline environments and possess the unique cellular enzymatic machinery, involved in balancing the osmotic stress, allowing them to thrive in high salinity. A ″Salt-in″ strategy based on accumulating concentrations of KCl equal to those of NaCl and the presence of specific halophilic enzymes play a key role in their survival (3).

Salt-rich environments are the basis of halotherapy, a treatment where patients inhale air from pollutants-free salt-mines (4). Halotherapy may have a beneficial effect on the quality of life of asthmatics (5). Polish salt-mines, where halotherapy is performed, are health resorts in Bochnia and Wieliczka (6). However, in addition to salt-saturated air, the air of these mines also contain various micro-organisms, including halophilic Archaea (7–9), such as *Halorhabdus rudnickae*, isolated from the Wieliczka salt-mine (9).

Archaeal species, including halophiles, have also been identified in the human microbiome (10). They have been detected in human stool samples (11) and in the intestinal mucosa of inflammatory bowel disease patients (12). However, so far, no pathogenic strain of halophiles has been identified. While the interactions of other types of Archaea, such as Methanoarchaea, with innate immune cells have been studied in mice and humans (13–15), until now there are no reports describing the interaction between halophiles and human immune cells.

As dendritic cells (DCs) are the prime professional antigen-presenting cells (16), which link innate and adaptive immunity (16, 17) and control T-cell responses by the delivery of co-stimulatory signals and cytokines (18), we examined here the effect of the halophilic archaeal strains *Hrd. rudnickae 64, Hrd. rudnickae 66* and *Natrinema salaciae* on human monocyte-derived DCs (DCs) and T cells. We examined the expression of surface markers on DCs and the cytokine production by DCs, as well as by autologous CD4^+^ T cells, naïve CD45RA^+^CD4^+^ T and memory CD45RO^+^CD4^+^ T cells, co-cultured with halophile-stimulated DCs. The main conclusion of this paper is that the two studied species of halophilic archaeons are recognized by human DCs and are able to induce a balanced Th1/Th2 cytokine response.

## Results

### Halophilic Archaea Induced Expression of CD86, CD80 And CD83 On Human DCs

**Figure 1:**
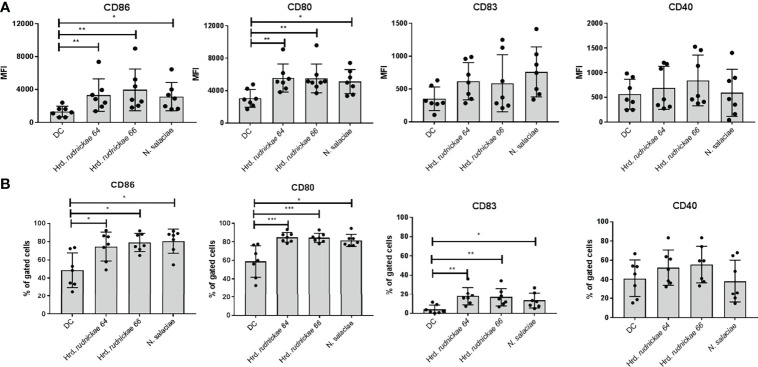
Surface marker expression on human DCs stimulated with halophilic archaea. Human DCs from healthy blood donors were stimulated for 24h either with *Hrd. rudnickae 64*, *Hrd. rudnickae 66*, *N. salaciae* or were left unstimulated (DC). Fluorescence intensity is expressed as MFI (once for each donor) of CD86, CD80, CD83 and CD40 surface expression on DC, from which the MFI obtained with an isotype-matched antibody was subtracted **(A)** and the percentages of positive cells with the CD86, CD80, CD83 and CD40 expression on DC surface **(B)** were calculated. Data shown represent the means ± SD of 7 independent donors. Statistical analyses were performed using the Mann–Whitney U test for unpaired data. *p < 0.05; **p < 0.01; ***p < 0.001 versus control (unstimulated DC).

To determine the effect of halophilic archaea on human DCs the cells were stimulated with *Hrd. rudnickae 64, Hrd. rudnickae 66* or *N. salaciae* at MOIs of 1:1 (1x10^6^ DCs: 1x10^6^ archaea) for 24h. Expression of surface markers was evaluated by flow cytometry. In preliminary experiments we have tested different MOI, 0.1:1, 1:1 and 1:10 and found that for most markers the MOI of 1:1 was the most appropriate (**Supplementary Figures S1**, **S2**), with respect to both the fluorescence intensity and frequency. In all subsequent experiments we therefore used the MOI of 1:1. Stimulation with *Hrd. rudnickae 64, Hrd. rudnickae 66* or *N. salaciae* resulted in significantly increased expression of CD86 and CD80 on the surface of DCs in comparison to unstimulated cells (**Supplementary Figure S3** and **Figure 1A**). Incubation of the DCs with these halophiles also resulted in a significantly increased percentage of DCs expressing CD86 and CD80 in comparison to non-stimulated cells (**Figure 1B**). DCs stimulated with halophilic archaea also showed significantly higher percentage of cells expressing CD83 than unstimulated cells (**Figure 1B**), while there was a trend only for increased mean fluorescence intensity (**Figure 1A**). In contrast to these surface markers, DCs stimulated with the halophiles did not appear to increase the expression of CD40 (**Figure 1**). As expected for the controls, DCs stimulated with *E. coli* LPS significantly increased the expression of all four surface markers [data not shown]. No significant changes in HLA-DR, DC-SIGN, TLR2 and TLR4 expression on DCs stimulated with halophilic archaea was observed (**Supplementary Figure S4**).

### DC Stimulated With Halophilic Archaea Produced IL-12p40, IL-10 and TNF-α but Not IL-23 and IL-12p70

**Figure 2:**
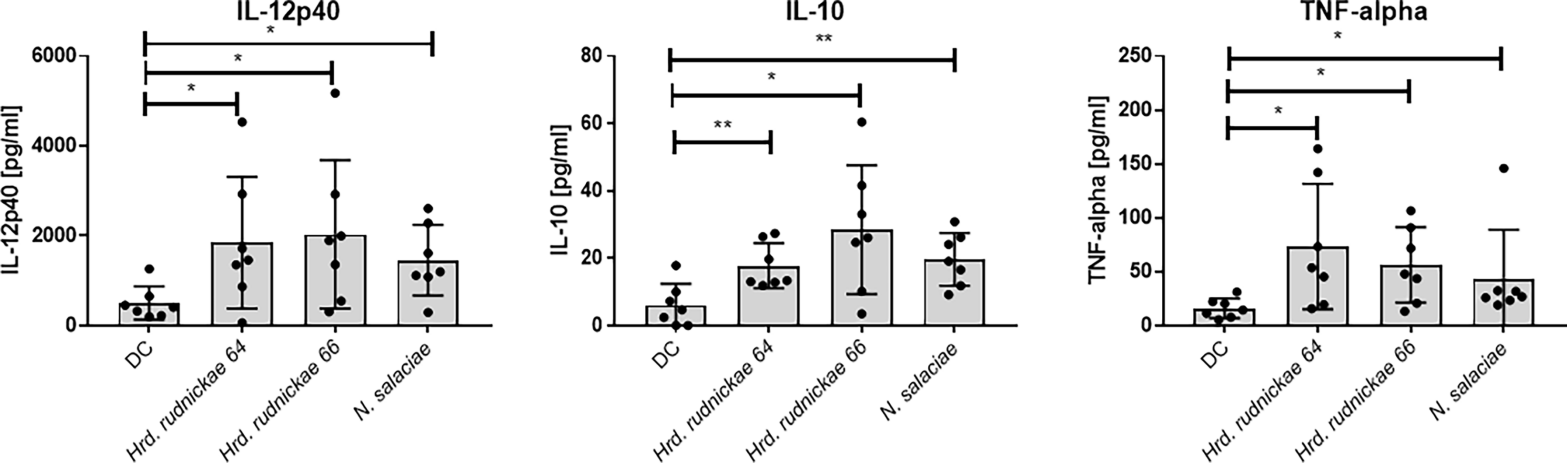
Cytokine secretion by halophile-stimulated DCs. Human DCs were stimulated either with *Hrd. rudnickae 64*, *Hrd. rudnickae 66*, *N. salaciae* or were left unstimulated (DC) for 24h. The levels of IL-12p40, IL-10 and TNF-α secretion by stimulated DCs were measured in duplicates by ELISA. Data shown represent the means ± SD of 7 donors. Statistical analyses were performed using the Mann–Whitney U test for unpaired data. *p < 0.05; **p < 0.01 versus control (unstimulated DC).

To investigate further the effect of DCs stimulation with *Hrd. rudnickae 64, Hrd. rudnickae 66* or *N. salaciae*, cytokines levels were measured by ELISA. After 24h of stimulation with halophilic archaea at a MOI of 1:1, cytokine secretion was measured by ELISA in duplicates. Stimulated DCs showed significantly higher production of IL-12p40, IL-10 and TNF-α than unstimulated cells (**Figure 2**), but did not induce IL-23, no IL-12p70 production (**Supplementary Figure S5**). For all analyzed cytokines, stimulation with *E. coli* LPS resulted in significantly higher production than unstimulated cells [data not shown].

### Autologous CD4^+^ T Cells Co-cultured With Halophilic Archaea-Stimulated DCs Produced IFN-γ and IL-13

As DCs are able to orient the immune responses towards Th1, Th2 or Th17 profiles, we investigated whether the DCs stimulated by the halophilic archaea may orient the T cell responses preferably to one or the other profile. Therefore, autologous CD4^+^ T cells were co-cultured with the stimulated DCs, and the production of IFN-γ, IL-13 and IL-17A, as a Th1, Th2 and Th17 cytokine representative, respectively, in the DC-T cell co-cultures were determined. DCs were thus stimulated for 24h with *Hrd. rudnickae 64, Hrd. rudnickae 66* or *N. salaciae* at MOIs of 1:1. Autologous CD4^+^ T cells were then added in a 1:10 ratio (1x10^5^ DCs: 1x10^6^ CD4^+^ T cells). This ratio was chosen based on previous studies using different stimuli in our laboratory (19) and by others (20–22). After 96h of co-culture the cytokine levels were measured by ELISA in duplicates.

**Figure 3:**
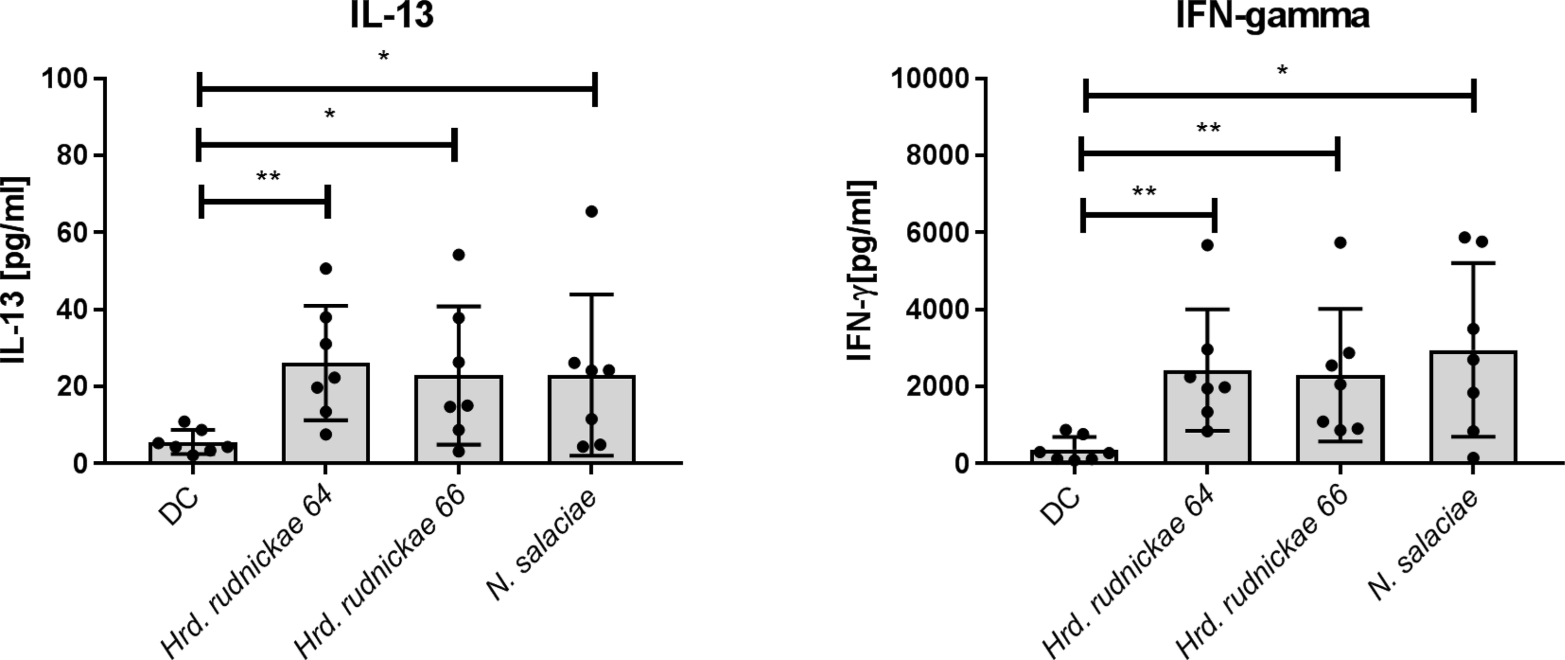
Cytokine secretion by CD4^+^ T cells co-cultured with halophile-stimulated DCs. Secretion of IFN-γ and IL-13 by human CD4^+^ T cells following 96 h co-culture with *Hrd. rudnickae* 64-, *Hrd. rudnickae* 66- or *N. salaciae-*pulsed autologous DCs (ratio DCs:T cells, 1:10) was measured in duplicates by ELISA. Data shown represent the means ± SD of 7 donors. Statistical analyses were performed using the Mann–Whitney U test for unpaired data. *p < 0.05; **p < 0.01 versus control (unstimulated DC).

Autologous CD4^+^ T cells produced significantly higher amounts of both IFN-γ and IL-13 (**Figure 3**) in response to *Hrd. rudnickae 64*-*, Hrd. rudnickae 66*- or *N. salaciae-*stimulated DCs in comparison to T cells co-cultured with unstimulated DCs but did not stimulate IL-17A production (**Supplementary Figure S6**). As expected, for all analyzed cytokines, stimulation with *E. coli* LPS resulted in significantly higher production of these cytokines than for control cells (data not shown).

### Naïve but not Memory T Cells Produced IFN-γ When Co-Cocultured With Halophile-Stimulated DCs

To determine whether the IFN-γ and IL-13 cytokine responses came from naïve or from memory CD4^+^ T cells, DCs were stimulated for 24h with *Hrd. rudnickae 64, Hrd. rudnickae 66* or *N. salaciae*, and autologous naïve or memory CD4^+^ T cells were then added in a 1:10 ratio. After 96h of co-culture the cytokine levels were determined by ELISA in duplicates.

**Figure 4:**
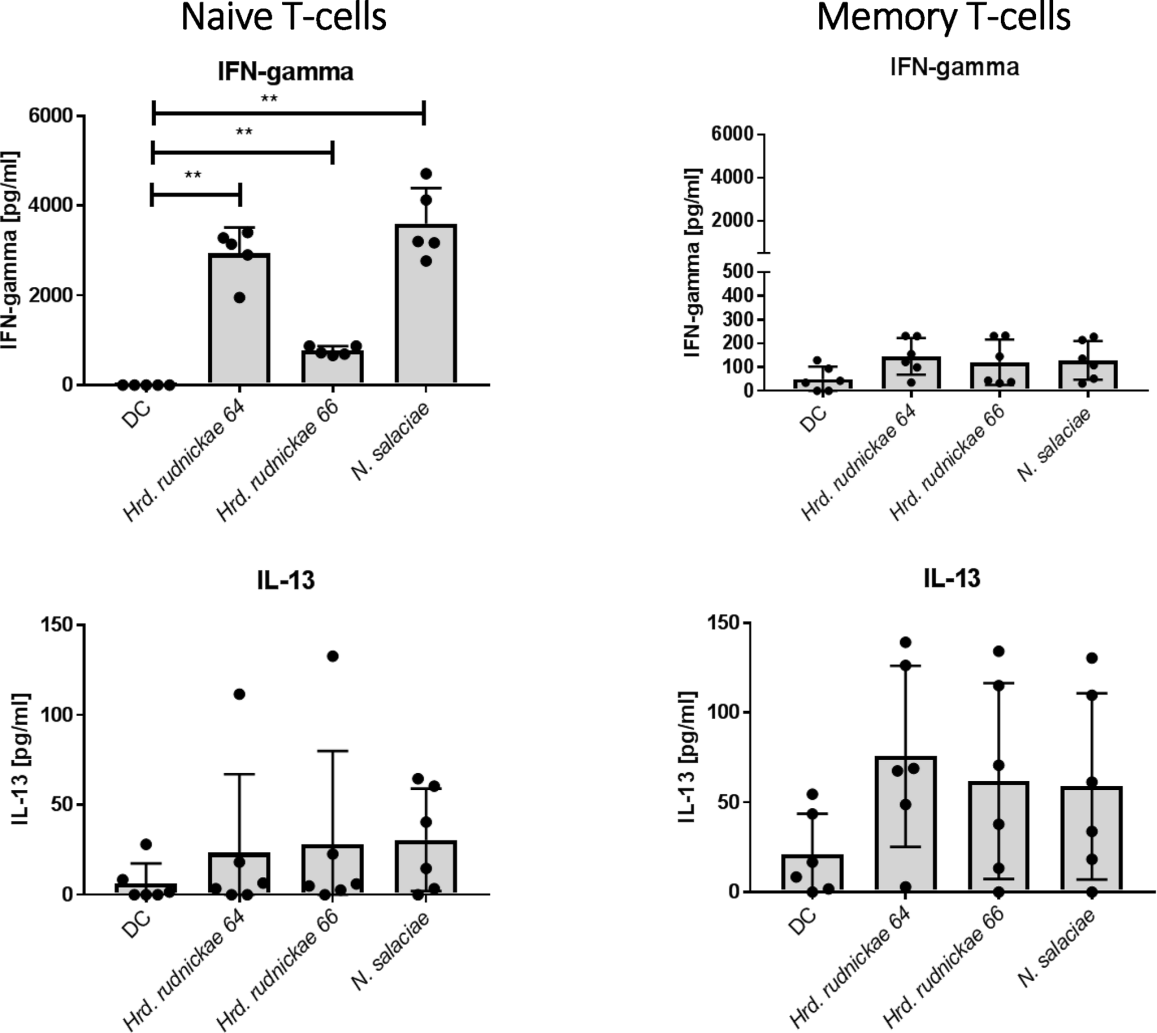
Cytokine secretion by naïve and memory CD4^+^ T cells co-cultured with halophile-stimulated DCs. Secretion of IFN-γ and IL-13 by human naïve (left panels) and memory (right panels) T cells following 96 h coculture with *Hrd. rudnickae 64-*, *Hrd. rudnickae 66-* or *N. salaciae-*pulsed autologous DCs (ratio DCs/T cells, 1:10) was measured in duplicates by ELISA. Data shown represent the means ± SD of 7 donors. Statistical analyses were performed using the Mann–Whitney U test for unpaired data. **p < 0.01 versus control (unstimulated DC).

As shown in **Figure 4**, naïve CD4^+^ T cells co-cultured with halophile-stimulated DCs were the main CD4^+^ T cell subsets producing significantly higher amounts of IFN-γ in comparison to T cells incubated with unstimulated DCs. Only minimal amounts of IFN-γ were produced by memory CD4^+^ T cells co-cultured with halophile-stimulated DCs, and no significant differences were observed compared to memory CD4^+^ T cells co-cultured with non-stimulated DCs (**Figure 4**). For IL-13 a trend of increased production was noted by both naïve and memory CD4^+^ T cells, which, however, did not reach statistically significance compared to co-cultures with untreated DCs.

## Discussion

The human body is exposed to halophilic archaea present in environments such as salt mines, in the human intestine and on skin (23, 24), as well as in salty food, including salted fish, ham and sausages (25). In this study we focused on the effect of two halophilic archaeal species, *Hrd. rudnickae* and *N. salaciae* on the human immune system. Both halophilic species activated DCs by the upregulation of the surface markers CD80, CD86 and CD83, while CD40, HLA-DR and DC-SIGN and TLR 2 and 4 were not upregulated by *in vitro* incubation of the DCs with these halophilic species. Furthermore, both species triggered the secretion of IL-12p40, IL-10 and TNF-α, suggesting a balanced immune activation. IL-23 was not induced by the two halophilic species. Since the two halophiles induced IL-10 by human DCs their effect may be anti-inflammatory, which is consistent with halotherapy having a beneficial effect for asthmatics (5), patients with chronic bronchitis, chronic obstructive bronchopneumopathy (4) and cystic fibrosis (26). It has also been proposed as an adjunct to conventional treatment of sub-obstructive adenotonsillar syndrome (27). By their immunoregulatory properties halophilic archaea may thus potentially contribute to the anti-inflammatory processes during halotherapy. Although this study was performed on species found in either a salt mine (*Hrd. rudnickae*) or the Mediterranean Sea (*N. salaciae*), the fact that they induce the same type of immune activation, although representing distinct genera, suggests that our observations may be broadly applicable to halophilic archaea.

Immunomodulatory properties of halophilic archaea have so far not yet been investigated, but phylogenetically closely related Methanobacteriales have been shown to also activate the mammalian immune system. *Methanospera stadtmanii* was found to trigger the accumulation of myeloid DCs in aerosol-exposed mice, while another methanogenic archaeal species, *Methanobrevibacter smithii*, was less potent in inducing myeloid DCs accumulation in the airways (13). Cytokine responses and surface marker expression were not measured in that study. However, a subsequent study showed that both species were able to induce the release the TNF-α by human PBMCs *in vitro*, but *M. stadtmanii* induced a four-fold higher response than *M*. *smithii* (14). Interestingly, *M. stadtmanii* was also isolated with a higher prevalence from stools of inflammatory bowel disease patients than of controls, unlike *M. smithii*, which was isolated at similar frequencies in both groups, suggesting a potential role of *M. stadtmanii* in the pathogenesis of inflammatory bowel disease. Increased TNF-α and IL-1ß secretion by human DCs stimulated with *M. stadtmanii* over *M. smithii* was confirmed by Bang et al. (15). In addition, these authors analysed the DC expression of CD86 and found that the expression of this surface marker was higher on DCs stimulated with *M. stadtmanii* than with *M. smithii*. The production of IL-10 induced by these archaea was not investigated. In contrast to these studies, we did not detect differences between the two species studied here with respect to DC activation. Furthermore, halophilic archaea have not been associated so far with any disease manifestation, possibly because of their ability to induce a balanced pro- and anti-inflammatory innate response, as shown here. On the other hand, halophilic archaea have been detected in human samples much less frequently than methanogenic archaea, which may be one of the reasons why the former have attracted less attention than the later. Nevertheless, halophilic archaea have been isolated from biopsies and faecal samples of inflammatory bowel disease patients (12). However, they have also been detected in intestine of healthy subjects (25, 28), although a recent study showed that halophilic archaea are more frequently present in stool samples from colorectal cancer patients than in healthy subjects (29).

Since DCs are crucial for the induction of adaptive immune responses and are key players in the orientation of T cell responses, we also investigated whether the activation of human DCs by halophilic archaea may have a downstream effect on T cells. When autologous CD4^+^ T cells were co-cultured with *Hrd. rudnickae*- or *N. salaciae*-activated DCs, both IFN-γ and IL-13 expression was induced, indicating a mixed Th1 and Th2 activation. In this study we have used the autologous T cells that were frozen during DC differentiation and thawed before incubation with the DCs rather than freshly isolated T cells. We had to use frozen and thawed T cells because it was legally not possible to bleed the donors again six days after the initial blood draw. We tested the viability of the T cells after freezing and thawing by trypan blue exclusion, although this method does not inform about apoptosis or functionality of the T cells. However, the fact that the frozen/thawed T cells secrete Th1- and Th2-type cytokines indicates that they remained functional after the freezing/thawing cycle. Although similar studies have not been performed with other archaea yet, Blais-Lecours et al. (13) have previously shown that the number of lymphocytes were elevated in the bronchiolar lavage fluids of mice intranasally instilled with *M. smithii* or *M. stadtmanae* in a dose-dependent manner. These lymphocytes were essentially CD4^+^ T cells and CD19^+^ B cells. Both methanogenic archaea also induced antigen-specific IgG in the serum of these mice. Serum IgG against *M. smithii*, as well as against other methanogenic archaea, such as *Methanobrevibacter oralis*, were also detected in human patients with periodontitis (30), indicating that they are also immunogenic in humans. This was later confirmed by a study showing that inflammatory bowel disease patients that had detectable levels of *M. stadtmanae* in their stool, as evidenced by qPCR, also mounted specific serum IgG responses against this microorganism (14). This was not the case in patients for which *M. smithii* had been identified in their stools, again showing a difference in the two methanogenic archaea. However, T cell responses have not been investigated in this study. In our study, like for the DC activation, we found no significant differences in the CD4^+^ T cell responses between *Hrd. rudnickae* and *N. salaciae*. Both induced a comparable level of Th1 and Th2 responses by the T cells co-cultured with the halophile-stimulated DCs. The IFN-γ was almost exclusively produced by naïve T cells, while IL-13 might be produced by both naïve and memory CD4^+^ T cells.

To our knowledge, this is the first report on the effect of halophilic archaea on the maturation of human DCs and the subsequent orientation of CD4^+^ T cells. However, archaeosomes, which are unique liposomes composed of specific lipid extracts from halophilic archaea, almost exclusively from *Halobacterium salinarum*, have been produced and assessed as vaccine vehicles against various pathogens [for a recent review see (31)]. These liposomes have been shown to induce both antibody and T cell responses to the passenger antigens, implying that halophile compounds can activate antigen-presenting cells and trigger adaptive immunity. The ligands that trigger DC activation have not been identified yet. Archaea do not produce LPS and lack murein in the cell wall, but, instead, produce unusual ether lipids that do not engage TLR4 or TLR2. However, archaeal RNA may be a potent inducer of the TLR8- or TLR7-dependent NLRP3 inflammasome, as has been shown for *M. stadtmaniae* (32). It remains to be seen whether this also applies to halophilic archaea. In our study we have not characterized the compounds that may be responsible for the observed effects, which needs further analyses to identify potential DC ligands. Therefore, the mechanism used by halophilic archaea to activate DCs is not yet known and awaits identification of these ligands, which is currently being pursued in our laboratory.

Archaea are generally not considered as pathogenic organisms, although methanogenic archaea have been linked to various human diseases, such as colon cancer, inflammatory bowel disease and periodontitis (33). However, so far there has been no conclusive evidence that they may be causative agents of human diseases, and it is unlikely that they are bona fide pathogens (34). Instead, they rather seem to be part of the normal microbiota (35) and may even exert positive effects in the gastro-intestinal tract, by serving as a hydrogen sink (36).

In contrast to methanogenic archaea, halophilic archaea have only rarely been associated with disease in humans. A recent study with colorectal cancer patients found enrichment of halophilic archaea and depletion of methanogenic archaea in their stool compared healthy controls (29). However, this enrichment was concurrent with an enrichment of *Bacteroides fragilis*, a known oncogenic bacterium (37), and it remains to be determined whether archaea dysbiosis in favor of certain halophilic species contributes to the pathogenesis of colorectal cancer. On the other hand, beneficial effects of halophilic archaea have been illustrated and it has been proposed that they may be used to improve the safety and quality of salted fish, by decreasing the risk of histamine poisoning (38).

Much work needs thus to be done in order to determine the effects of halophilic archaea in human health and disease. Although essentially classified as extremophiles, halophilic archaea may also survive and replicate in mesophilic conditions, such as the human gut and skin. The fact that they can promote a balanced Th1/Th2 and pro-/anti-inflammatory immune response, as shown here for two species from different genera, suggests a potential for these organisms to re-establish the immune balance in patients suffering from immune dysfunction, such as inflammatory diseases, including allergic asthma.

## Materials and Methods

### Preparation of Halophilic Archaea

*Hrd. rudnickae 64 (WSM-64^T^=DSM29498^T^), Hrd. rudnickae 66 (WSM-66=DSM29499)* and *N. salaciae (DSM 25055^T^)* were kindly provided by Dr. Luciana Albuquerque and Prof. Milton S. da Costa from the University of Coimbra, Portugal (9, 39). *Hrd. rudnickae* strains are red pigmented, non-motile cocci-shaped Gram-negative facultative anaerobes with optimum growth at 40°C in 20% NaCl. The major polar lipids are phosphatidylglycerol (PG2), phosphatidylglycerol phosphate methyl ester (PGP-Me) and sulfated diglycosyl diether (S-DGD). Menaquinone MK-8 was the major respiratory quinone (9). *N. salaciae* is a Gram-negative, pleomorphic, non-motile procaryotic microorganism with optimal growth at 45°C in 15-20% NaCl. The major polar lipids are phosphatidylglycerol (PG1 and PG2), phosphatidylglycerol phosphate methyl ester (PGP-Me) and mannose-2,6-dissulfate (1 → 2)-glucose glycerol diether (S2 -DGD) (39). Halophilic strains were cultivated in 100 mL of *Halobacteria* medium (HBM) (5 g/L yeast extract, 5 g/L casamino acids, 1 g/L Na-glutamate, 2 g/L KCl, 3 g/L Na_3_-citrate, 20 g/L MgSO_4_ x 7 H_2_O, 36 mg/L FeCl_2_ x 4 H_2_O, 360 ng/L MnCl_2_ x 4 H_2_O) in 300 mL Erlenmeyer flasks. *Hrd. rudnickae 64* and *Hrd. rudnickae 66* were grown in halophilic medium with 20% of NaCl at 37°C for 48h, while *N. salaciae* was grown in halophilic medium with 15% of NaCl at 45°C for 48h.

Growth of halophilic cultures was monitored by the optical density measurements at 600 nm (OD_600_), and colony-forming unit numbers were determined by growth on HBM with 2% agar. For DC stimulation, archaea from 48h cultures at logarithmic growth were harvested. Halophiles were centrifuged at 4°C for 15 min at 4,500 x *g*. The pellets were washed by transferring them to new tubes, resuspended in 10 mL of 4°C PBS and centrifuged at 4°C for 15 min at 4,500 x *g*. Finally, cells were resuspended in complete RPMI.

### CD14^+^ and CD14^-^ Cell Purification From PBMCs by Magnetic Cell Sorting

Peripheral blood mononuclear cells (PBMCs) were isolated from anonymized, commercially available buffy coats of 7 healthy human blood donors (Regional Blood Donation Station, Lodz, Poland). The cells were diluted in PBS without Mg^2+^ and Ca^2+^, layered on a Ficoll-Paque PLUS, centrifuged 1,100 x *g* at 20°C for 30 min, collected and washed in cRPMI-1640 medium as described (40). Human blood monocytes were purified from PBMCs by positive immunomagnetic separation using anti-human CD14^+^ MACS Microbeads (Miltenyi Biotech, Germany) as described (19). Human CD4^+^ T cells were isolated from the CD14^-^ fraction by positive immunomagnetic separation on a LS column using anti-human CD4 MACS Microbeads (Miltenyi Biotech, Germany) in accordance with manufacturer specifications. CD4^+^ cells were frozen in freezing medium (45% cRPMI-1640, 45% FCS, 10% DMSO) and stored in -80°C until further use, as described (41). Frozen CD4^+^ T cells were used, as we had to wait for the generation of DCs by incubation of monocytes with GM-CSF and IL-4 during 6 days to be able to use autologous DCs and CD4^+^ T cells from the same donor at the same day. After thawing of the T cells, viability was assessed by trypan blue exclusion staining.

### Generation of Human DCs

The monocytes were suspended in RPMI-1640 supplemented with 100 U/ml penicillin, 0.1 mg/ml streptomycin, 2 mM L-glutamine (Gibco, Grand Island, NY) and enriched with 10% (v/v) FCS (heat inactivated; Cambrex, Belgium) (complete RPMI-1640, cRPMI-1640). The density was adjusted to 1 × 10^6^ cells/ml and the cells were seeded into 6-well flat-bottom plates (Falcon) and cultured for 6 days at 37°C, 5% CO_2_ in cRPMI-1640 medium in the presence of 10 ng/ml IL-4 and 25 ng/ml GM-CSF (R&D Systems, Minneapolis, MN) to allow cells to differentiate into DCs. The mean percentages of DCs obtained from monocytes isolated from buffy coats was 54.69%.

### DC Stimulation

Immature DCs at a density of 1x10^6^ cells/mL were incubated for 24 h (37°C, 5% CO_2_) with 1x10^6^ cells *Hrd. rudnickae 64*, *Hrd. rudnickae 66* or *N. salaciae.* Lipopolysaccharide (LPS) from *E. coli* O55:B5 (1 µg/mL) (Sigma) was used as a positive control, while DCs in cRPMI-1640 medium alone represented the negative control. Collected supernatants of the cultures were tested for IL-10, TNF-α, IL-12p40, IL-12p70 and IL-23 by ELISA using Diaclone’s kit (Immuniq, Zory, Poland). Detection sensitivities were: 5 pg/mL for IL-10, 8 pg/mL for TNF-α, 2.2 pg/ml for IL-12p70 and 20 pg/mL for IL-12p40 and IL-23.

### DC Surface Marker Analysis by Flow Cytometry

To determine the expression of DCs surface markers, the stimulated and unstimulated DCs were harvested from the 6-well plates using PBS/2mM EDTA, then washed in PBS and stained for 30 min. at 4°C with the following mAb (BectonDickinson): fluorescein isothiocyanate (FITC)-conjugated anti-CD86, (FITC)-conjugated anti-CD40, (FITC)-conjugated anti-DC-specific intracellular adhesion molecule-3 grabbing nonintegrin(DC-SIGN), (FITC)-conjugated anti-HLA-DR, or phycoerythrin (PE)-conjugated anti-CD80, (PE)-conjugated anti-CD83, (PE)-conjugated anti-TLR2, (PE)-conjugated anti-TLR4. An irrelevant isotype-matched mAb was used as control. Living cells were gated using forward and side scatter properties (FSC/SSC) and then using the specific markers indicated above. Data were analyzed using the FACS LSRII (BD) and FlowJo software. A minimum of 5,000 events was collected. Compensations were calculated using BD Compbeads with the automatic compensation program. Data are expressed as percentage of cells expressing the marker (% gated) and the mean fluorescence intensities (MFI), representing the molecular densities on the cell surface for each marker, after subtraction of the isotype control. The gating strategy and exemplary dot plots are shown in **Supplementary Figure S7**. One representative experiment showing the histograms of the expression of surface receptors on DCs is shown in **Supplementary Figure S3**.

### Co-Culture of DCs and Autologous CD4^+^ T Lymphocytes

The frozen CD4^+^ T lymphocytes were thawed and co-cultured with the autologous antigen-stimulated DCs at a ratio of 1x10^6^ DCs to 1x10^7^ T cells for 96h at 37°C with 5% CO_2_. Collected supernatants were tested for IFN-γ, IL-17A, and IL-13, production by ELISA using commercially available Diaclone’s kit (Immuniq, Zory, Poland). Detection sensitivities were: 5 pg/mL for IFN-γ, 2.3 pg/ml for IL-17A and 1.5 pg/mL for IL-13.

### Naive and Memory CD4^+^ T Cell Isolation

Naïve CD45RA^+^CD4^+^ T cells and memory CD45RO^+^CD4^+^ T cells were isolated from the eluted CD14^-^ cell fraction by using a naïve CD4^+^ T cell isolation kit and a memory CD4^+^ T cell isolation kit (Miltenyi Biotec), respectively, as described (19). Both cell fractions (purity>95%) were frozen at -80°C in freezing medium (45% cRPMI-1640, 45% FCS, 10% DMSO), as described (41), until used.

### Co-Culture of DCs and Autologous Naive and Memory CD4^+^ T Cells

The frozen naïve CD45RA^+^CD4^+^ T cells and memory CD45RO^+^CD4^+^ T lymphocytes were thawed and co-cultured with the autologous halophile-stimulated DCs at a ratio of 1x10^6^ DCs to 1x10^7^ T cells for 96h at 37°C with 5% CO_2_. Collected supernatants were tested for IFN-γ, and IL-13 production by ELISA using commercially available Diaclone’s kit (Immuniq, Zory, Poland).

### Statistical Analysis

Statistical analyses were performed with the GraphPad Prism 7 and STATISTICA 12.0 PL program. Data are expressed as mean ± SD. Differences between samples were analyzed by the analysis of variance Kruskal-Wallis non-parametric test and Mann-Whitney U test. *p* values ≤0.05 were considered significant.

## Data Availability Statement

The raw data supporting the conclusions of this article will be made available by the authors, without undue reservation.

## Ethics Statement

The studies involving human participants were reviewed and approved by Ethics Committee at the University of Lodz, Poland (3/KBBN-UŁ/II/2017). The patients/participants provided their written informed consent to participate in this study.

## Author Contributions

Conceptualization, MK-K and CL. Funding acquisition, KK and MK-K. Performed the experiments, KK and MK-K. Analyzed the data, MK-K, KK and CL. Contributed reagents/materials, KK, MK-K. Writing—original draft preparation, MK-K, CL and KK. Writing—review and editing, MK-K, CL. All authors reviewed and accepted the manuscript. All authors contributed to the article and approved the submitted version.

## Funding

This work was supported by the National Science Centre grant no 2017/27/N/NZ6/02850 awarded to KK and Visiting Research Fellow “Initiative of Excellence - Research University (IDUB) project, University of Lodz 2021 awarded to CL.

## Conflict of Interest

The authors declare that the research was conducted in the absence of any commercial or financial relationships that could be construed as a potential conflict of interest.

## Publisher’s Note

All claims expressed in this article are solely those of the authors and do not necessarily represent those of their affiliated organizations, or those of the publisher, the editors and the reviewers. Any product that may be evaluated in this article, or claim that may be made by its manufacturer, is not guaranteed or endorsed by the publisher.

## Supplementary Material

Supplementary Figure 1 CD86, CD80, CD83 and CD40 expression on human DCs stimulated with halophilic archaea at various MOI. Human DCs from blood donors were stimulated for 24h either with *Hrd. rudnickae 64, Hrd. rudnickae 66* or *N. salaciae* (MOI 0.1:1, 1:1, 1:10) or were left unstimulated (DC). Fluorescence intensity is expressed as MFI (once for each donor) of CD86, CD80, CD83 and CD40 surface expression on DC from which the MFI obtained with an isotype-matched antibody was subtracted **(A)**, and the percentages of positive cells with CD86, CD80, CD83 and CD40 expression on the DC surface was calculated **(B)**. Data shown represent the means ± SD of 3 healthy independent donors.

Supplementary Figure 2 IL-10, TNF-α, IL-12p40 production by DCs stimulated with halophilic archaea at various MOI. Human DCs were stimulated either with *Hrd. rudnickae 64*, *Hrd. rudnickae 66* or *N. salaciae* (MOI 0.1:1, 1:1, 1:10) or were left unstimulated (DC) for 24h. The levels of IL-10, TNF-α, IL-12p40 secretion by stimulated DCs were measured in duplicates by ELISA. Data shown represent the means ± SD of 3 donors.

Supplementary Figure 3 Surface marker CD86, CD80, CD83 and CD40 expression on human DCs stimulated with halophilic archaea. Human DCs from a healthy blood donor were stimulated for 24h either with *Hrd. rudnickae 64*, *Hrd. rudnickae 66*, *N. salaciae* or were left unstimulated (DC). Histograms represent the surface expression of CD86, CD80, CD83 and CD40 on DCs for one representative donor.

Supplementary Figure 4 Surface marker HLA-DR, DC-SIGN, TLR2 and TLR4 expression on human DCs stimulated with halophilic archaea. Human DCs from blood donors were stimulated for 24h either with *Hrd. rudnickae 64, Hrd. rudnickae 66* or *N. salaciae* or were left unstimulated (DC). Fluorescence intensity is expessed as MFI (once for each donor) of HLA-DR, DC-SIGN, TLR2 and TLR4 surface expression on DC from which the MFI obtained with an isotype-matched antibody was subtracted **(A)**, and the percentages of positive cells with HLA-DR, DC-SIGN, TLR2 and TLR4 expression on the DC surface was calculated **(B)**. Data shown represent the means ± SD of 7 healthy independent donors.

Supplementary Figure 5 IL-23 and IL-12p70 secretion by DCs stimulated with halophilic archaea. Human DCs were stimulated either with *Hrd. rudnickae 64*, *Hrd. rudnickae 66* or *N. salaciae* or were left unstimulated (DC) for 24h. The levels of IL-23, IL-12p70 secretion by stimulated DCs were measured in duplicates by ELISA. Data shown represent the means ± SD of 7 donors.

Supplementary Figure 6 IL-17A secretion by CD4^+^ T cells co-cultured with halophile-stimulated DCs. Secretion of IL-17A by human CD4^+^ T cells following 96 h co-culture with *Hrd. rudnickae* 64-, *Hrd. rudnickae* 66- or *N. salaciae-*pulsed autologous DCs (ratio DCs:T cells, 1:10) was measured in duplicates by ELISA. Data shown represent the means ± SD of 7 donors.

Supplementary Figure 7 Gating strategy for human DCs based on FSC/SSC criteria. Unstimulated DCs from blood donors were gated based on the forward (FSC) and side (SSC) scatter measurements **(A)**. Exemplary dot-plots showing human DCs stimulated for 24h with *Hrd. rudnickae 64* and stained for CD86-FITC **(B)** or CD80-PE **(C)** are shown for one representative donor.
