# Supplementary figures and images for: Halophilic Archaea Halorhabdus Rudnickae and Natrinema Salaciae Activate Human Dendritic Cells and Orient T Helper Cell Responses
Source: Front Immunol. 2022 May 26;13:833635. doi: 10.3389/fimmu.2022.833635 (PMC9204267; doi:10.3389/fimmu.2022.833635)

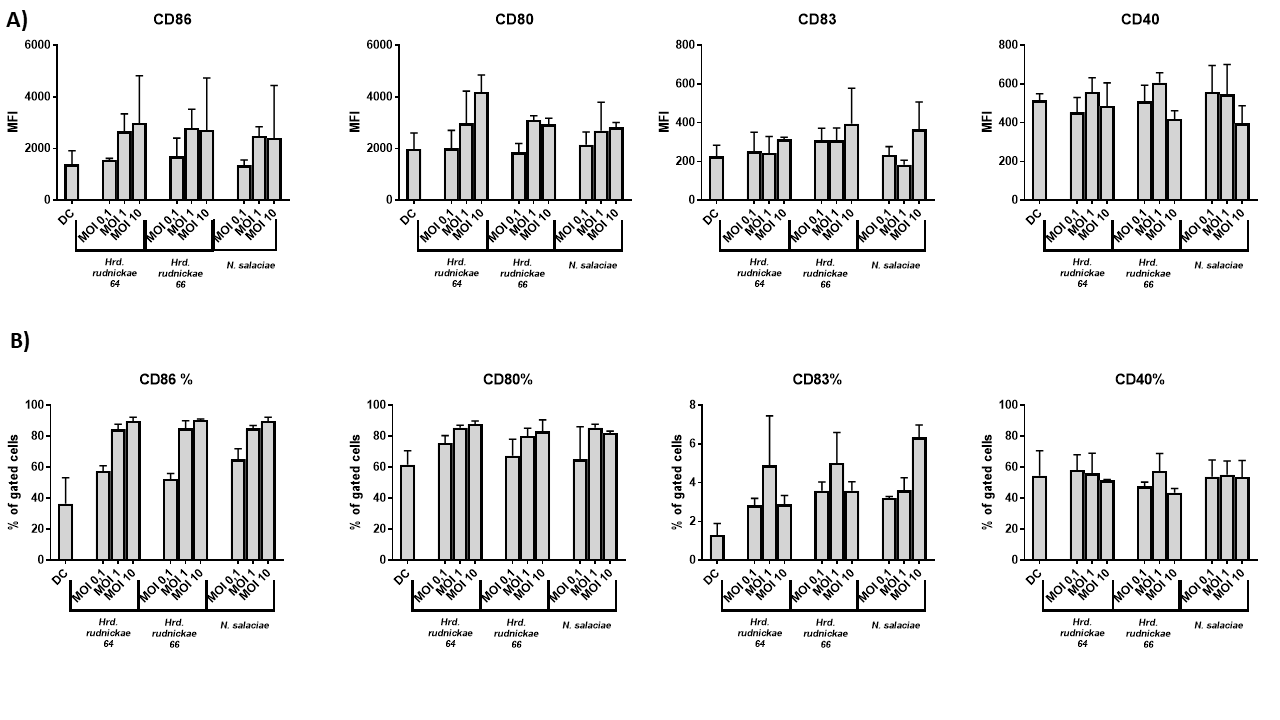

Supplement: Supplementary Figure 1 — CD86, CD80, CD83 and CD40 expression on human DCs stimulated with halophilic archaea at various MOI. Human DCs from blood donors were stimulated for 24h either with Hrd. rudnickae 64, Hrd. rudnickae 66 or N. salaciae (MOI 0.1:1, 1:1, 1:10) or were left unstimulated (DC). Fluorescence intensity is expressed as MFI (once for each donor) of CD86, CD80, CD83 and CD40 surface expression on DC from which the MFI obtained with an isotype-matched antibody was subtracted (A), and the percentages of positive cells with CD86, CD80, CD83 and CD40 expression on the DC surface was calculated (B). Data shown represent the means ± SD of 3 healthy independent donors. [file Image_1.tif]

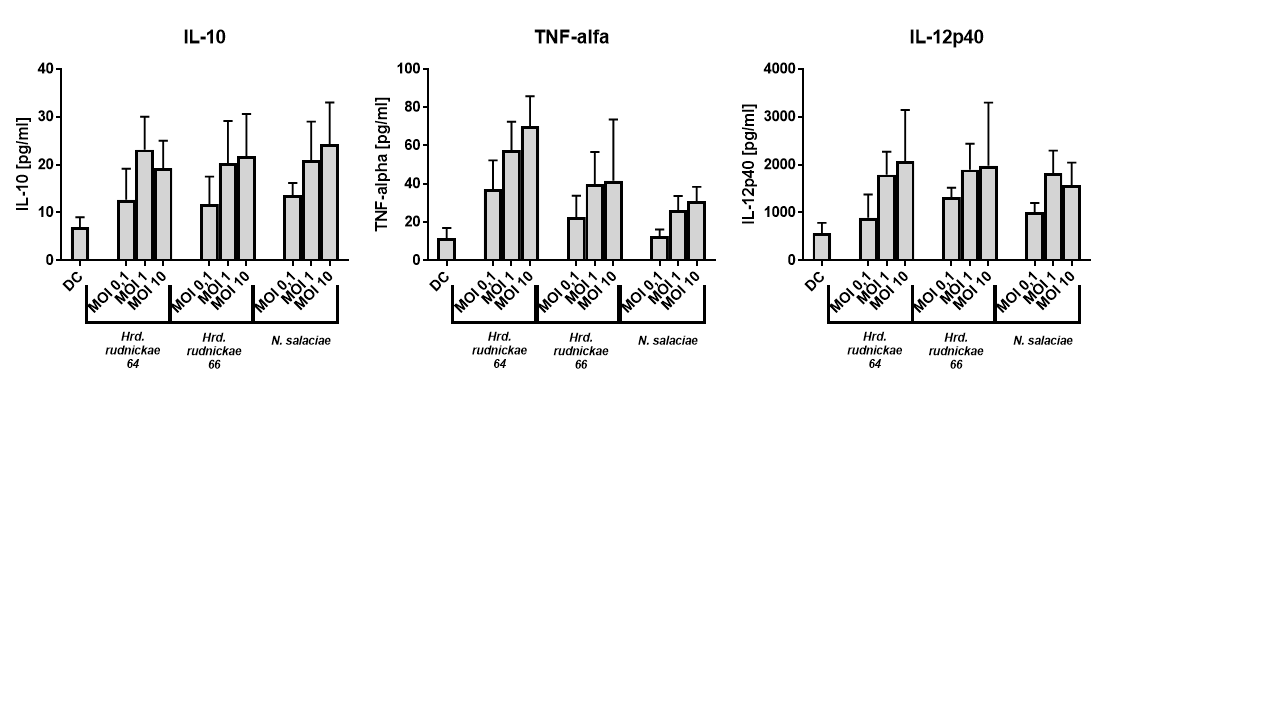

Supplement: Supplementary Figure 2 — IL-10, TNF-α, IL-12p40 production by DCs stimulated with halophilic archaea at various MOI. Human DCs were stimulated either with Hrd. rudnickae 64, Hrd. rudnickae 66 or N. salaciae (MOI 0.1:1, 1:1, 1:10) or were left unstimulated (DC) for 24h. The levels of IL-10, TNF-α, IL-12p40 secretion by stimulated DCs were measured in duplicates by ELISA. Data shown represent the means ± SD of 3 donors. [file Image_2.tif]

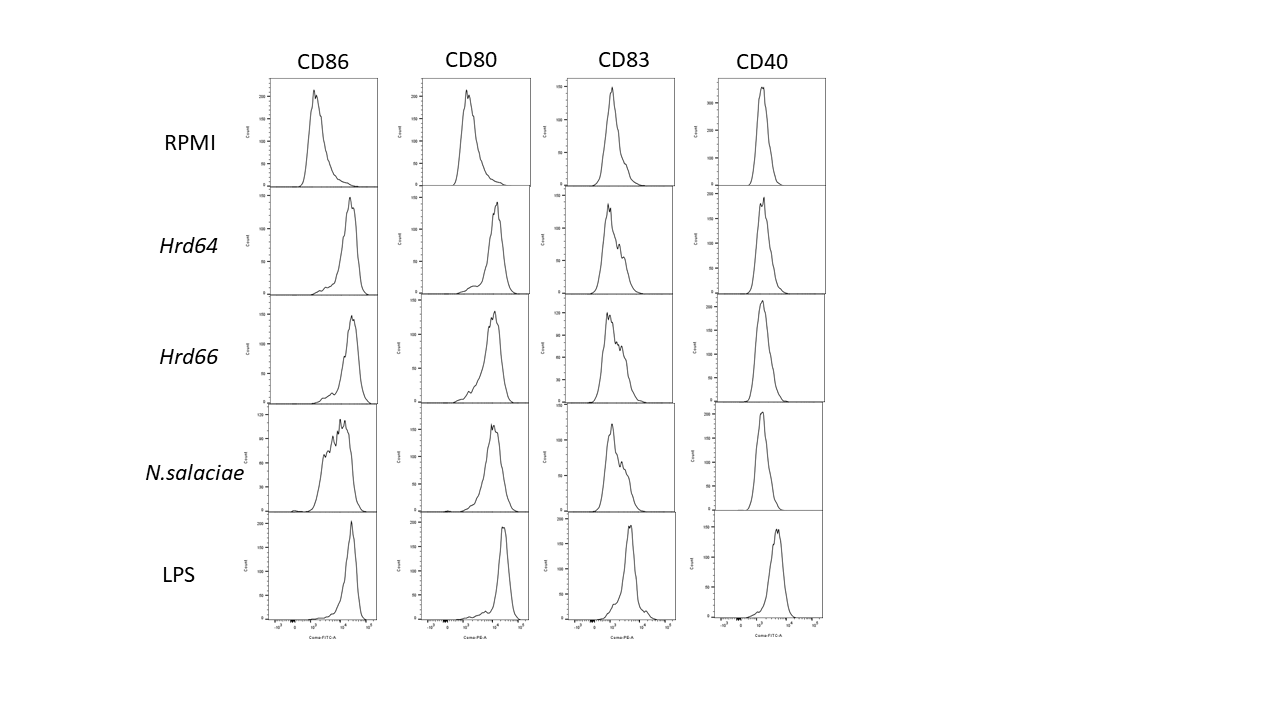

Supplement: Supplementary Figure 3 — Surface marker CD86, CD80, CD83 and CD40 expression on human DCs stimulated with halophilic archaea. Human DCs from a healthy blood donor were stimulated for 24h either with Hrd. rudnickae 64, Hrd. rudnickae 66, N. salaciae or were left unstimulated (DC). Histograms represent the surface expression of CD86, CD80, CD83 and CD40 on DCs for one representative donor. [file Image_3.tif]

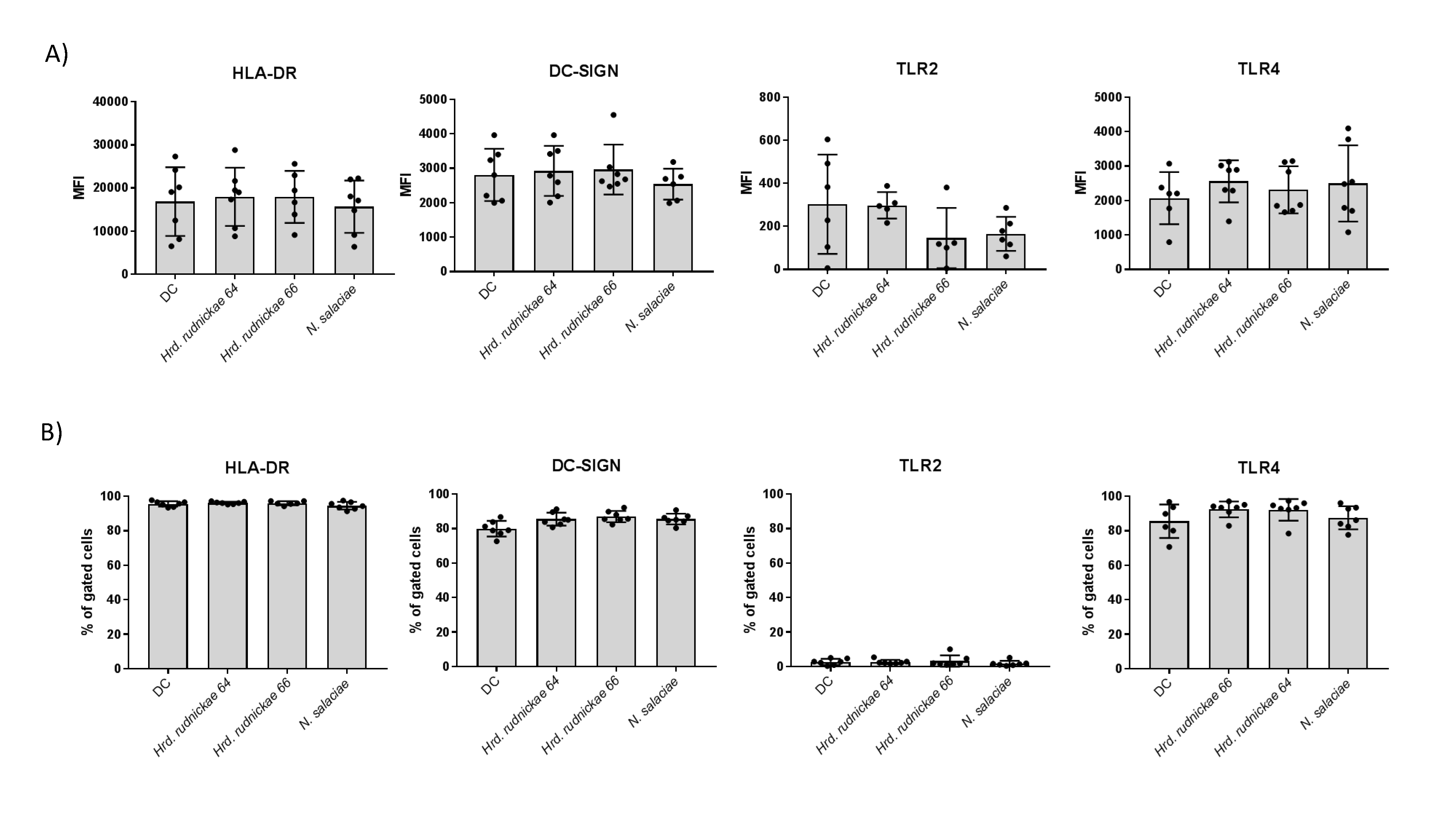

Supplement: Supplementary Figure 4 — Surface marker HLA-DR, DC-SIGN, TLR2 and TLR4 expression on human DCs stimulated with halophilic archaea. Human DCs from blood donors were stimulated for 24h either with Hrd. rudnickae 64, Hrd. rudnickae 66 or N. salaciae or were left unstimulated (DC). Fluorescence intensity is expessed as MFI (once for each donor) of HLA-DR, DC-SIGN, TLR2 and TLR4 surface expression on DC from which the MFI obtained with an isotype-matched antibody was subtracted (A), and the percentages of positive cells with HLA-DR, DC-SIGN, TLR2 and TLR4 expression on the DC surface was calculated (B). Data shown represent the means ± SD of 7 healthy independent donors. [file Image_4.tif]

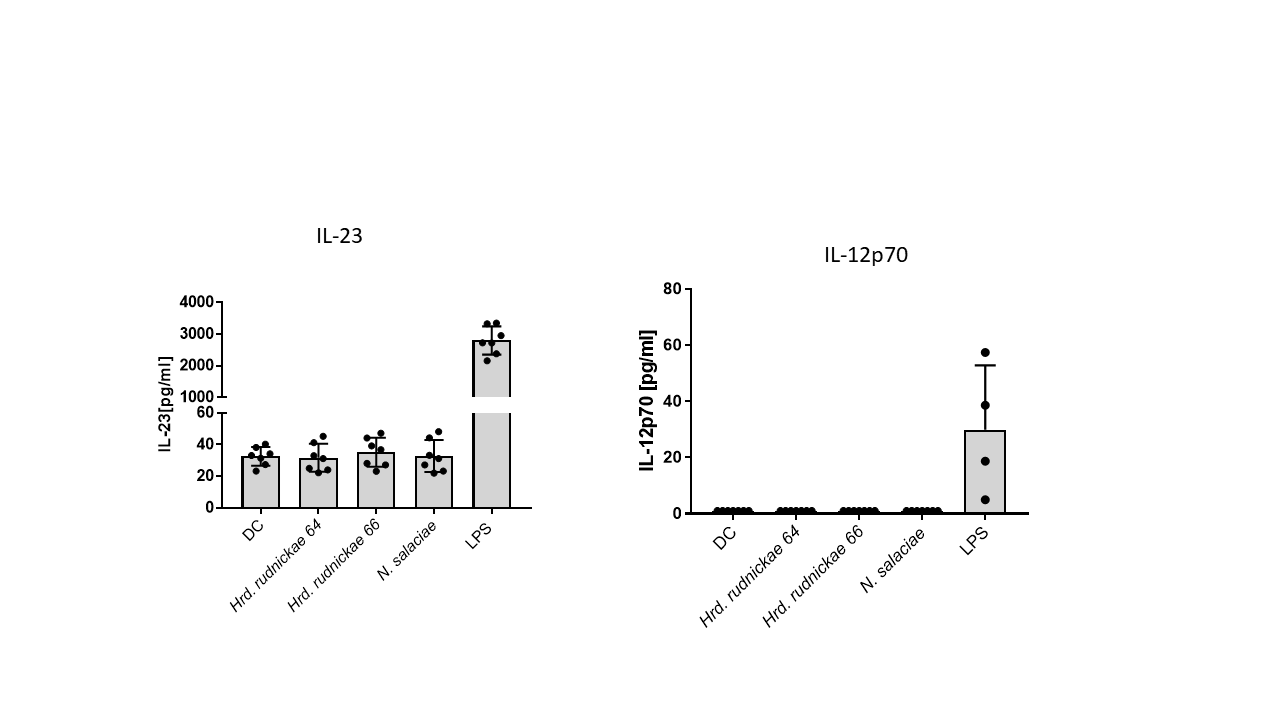

Supplement: Supplementary Figure 5 — IL-23 and IL-12p70 secretion by DCs stimulated with halophilic archaea. Human DCs were stimulated either with Hrd. rudnickae 64, Hrd. rudnickae 66 or N. salaciae or were left unstimulated (DC) for 24h. The levels of IL-23, IL-12p70 secretion by stimulated DCs were measured in duplicates by ELISA. Data shown represent the means ± SD of 7 donors. [file Image_5.tif]

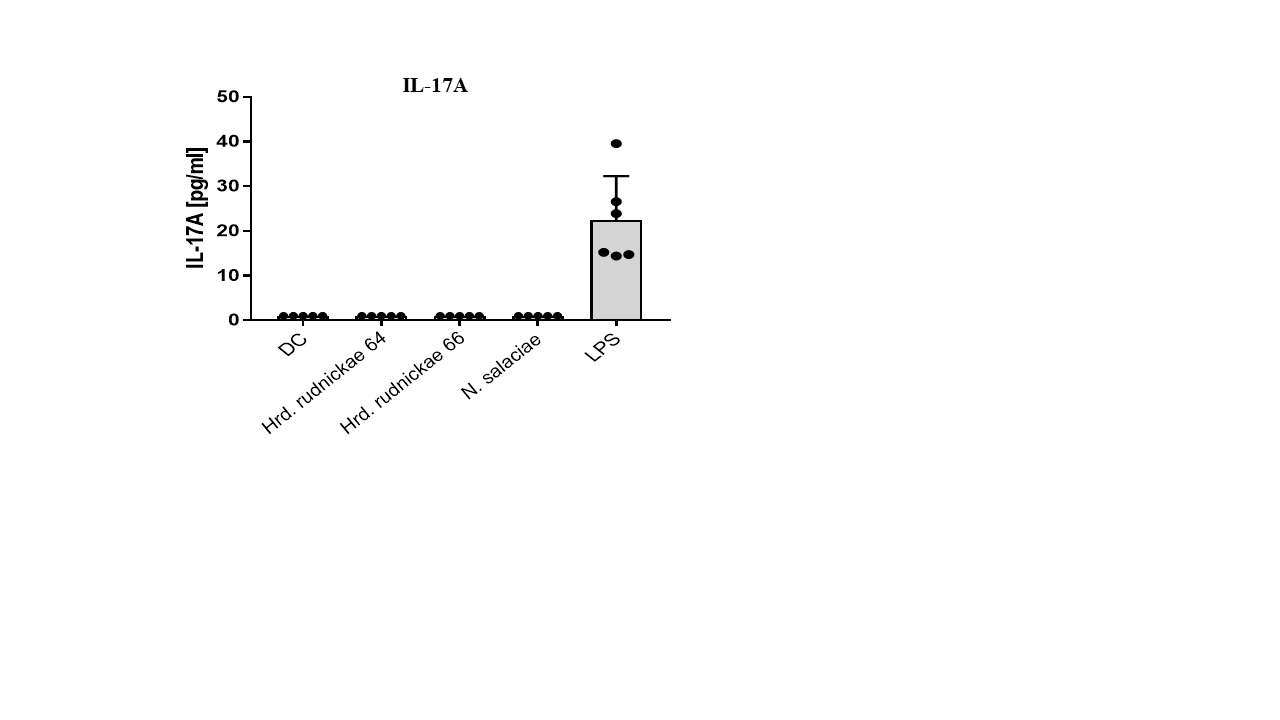

Supplement: Supplementary Figure 6 — IL-17A secretion by CD4+ T cells co-cultured with halophile-stimulated DCs. Secretion of IL-17A by human CD4+ T cells following 96 h co-culture with Hrd. rudnickae 64-, Hrd. rudnickae 66- or N. salaciae-pulsed autologous DCs (ratio DCs:T cells, 1:10) was measured in duplicates by ELISA. Data shown represent the means ± SD of 7 donors. [file Image_6.tif]

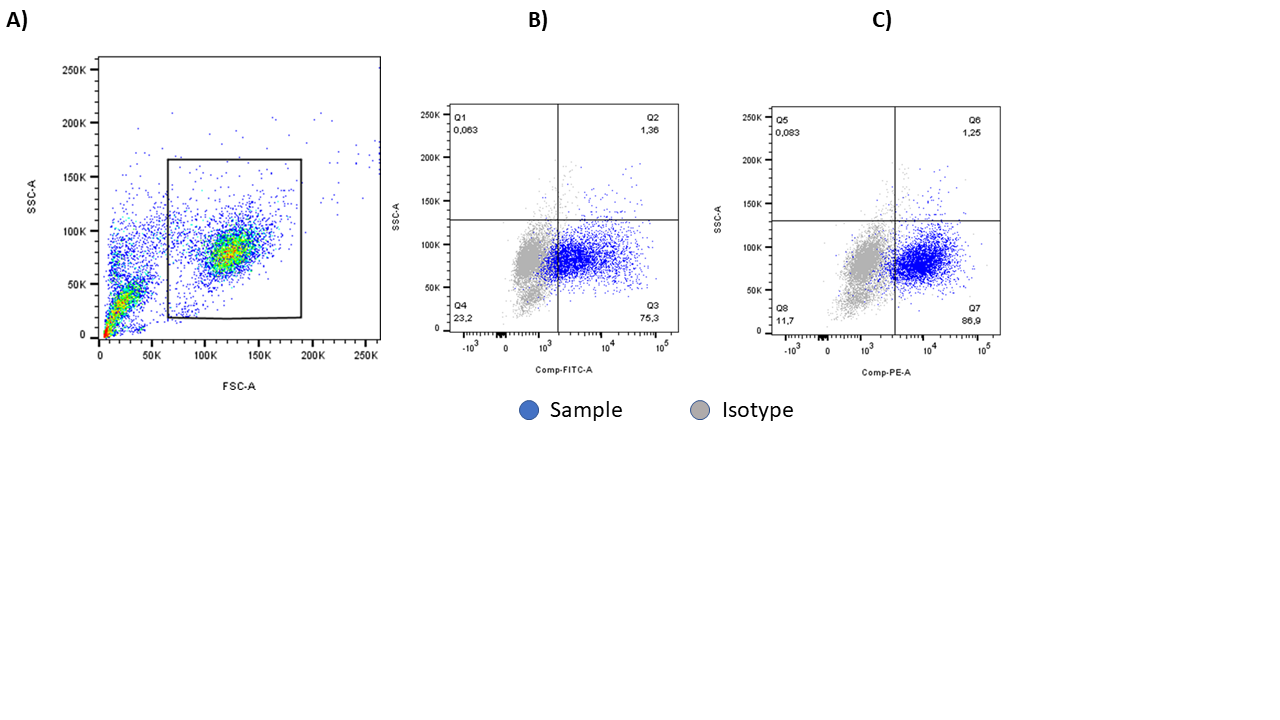

Supplement: Supplementary Figure 7 — Gating strategy for human DCs based on FSC/SSC criteria. Unstimulated DCs from blood donors were gated based on the forward (FSC) and side (SSC) scatter measurements (A). Exemplary dot-plots showing human DCs stimulated for 24h with Hrd. rudnickae 64 and stained for CD86-FITC (B) or CD80-PE (C) are shown for one representative donor. [file Image_7.tif]
